# Long Noncoding RNA HOTAIR Is Associated with Motility, Invasion, and Metastatic Potential of Metastatic Melanoma

**DOI:** 10.1155/2013/251098

**Published:** 2013-06-05

**Authors:** Lihua Tang, Wei Zhang, Bing Su, Bo Yu

**Affiliations:** ^1^The Affiliated Shenzhen Hospital, Anhui Medical University, Hefei, Anhui 230001, China; ^2^Biomedical Research Institute, Shenzhen PKU-HKUST Medical Center, Shenzhen, Guangdong 518036, China; ^3^Shenzhen Key Lab for Translational Medicine of Dermatology, Shenzhen PKU-HKUST Medical Center, Shenzhen, Guangdong 518036, China; ^4^Shenzhen Key Discipline of Dermatology, Shenzhen Hospital, Peking University, Shenzhen, Guangdong 518036, China; ^5^Department of Dermatology, Shenzhen Hospital, Peking University, Shenzhen, Guangdong 518036, China

## Abstract

Metastatic melanoma, the primary cause of skin cancer-related death, warrants new therapeutic approaches that target the regulatory machinery at molecular level. While long noncoding RNAs (lncRNAs) are dysregulated in a number of cancer types, limited data are available on the expression and function of lncRNAs in melanoma metastasis. The primary objective of this study was to investigate the role of 6 metastasis-related lncRNAs in pairs of primary melanoma and matched lymph node metastatic tissues. Among the tested lncRNAs, HOTAIR was the most highly expressed in lymph node metastasis. The role of HOTAIR in melanoma cell motility and invasion was further evaluated by knocking down HOTAIR with siRNAs. Knockdown of HOTAIR resulted in the reduction of motility and invasion of human melanoma cell line A375, as assessed by wound healing assay and Matrigel-based invasion assay. siHOTAIR also suppressed the degradation of gelatin matrix, suggesting that HOTAIR promotes gelatinase activity. Together, our study shows that HOTAIR is overexpressed in metastatic tissue, which is associated with the ability of HOTAIR to promote melanoma cell motility and invasion. These data indicate that lncRNAs may be involved in the metastasis of melanoma and provide support for further evaluation of lncRNAs in melanoma.

## 1. Introduction

Although the incidence of malignant melanoma accounts for only approximately 1% of cutaneous tumors, it is among the few cancers with a remarkably high mortality rate. This is mainly due to its high potential to metastasize to the vital organs such as lungs, liver, and brain. Approximately 80% of skin cancer-related mortality is due to melanoma (http://www.chinaswzl.com/cancer/hsslby/2493.html).

The worldwide incidence of melanoma is increasing [1]. Statistical analysis of the melanoma cases collected in recent years in our department shows that the incidence of melanoma is much higher than the Chinese national average and is still increasing, especially in the younger population in southern industrial cities in China. This phenomenon may be caused by complex environmental factors and long sun exposure (Yu B, unpublished data).

Noncoding RNAs (ncRNAs) are emerging as new regulators in the cancer paradigm. They have demonstrated potential roles in both oncogenic and tumor suppressive pathways [2, 3]. ncRNAs are largely grouped into two major classes based on transcript sizes: small ncRNAs (<200 kb) and long ncRNAs (lncRNAs) (>200 kb) [4, 5]. Small ncRNAs include a broad range of well-known and newly discovered RNA species, with many being associated with 5′ or 3′ regions of genes. This class includes the well-documented miRNAs. It has been widely reported that cancer-specific miRNAs can be detected in the blood, sputum, and urine of cancer patients and serve as diagnostic and prognostic markers [6–8].

LncRNAs, ranging from 200 nucleotides to over 10 kb, are abundantly transcribed by the mammalian genome [9, 10]. LncRNAs have been found to be dysregulated in a wide range of human diseases and disorders, including various cancers. For example, PCGEM [11] and DD3 [12] are overexpressed in prostate cancer as compared to adjacent normal prostate tissue, implicating a role for these lncRNAs in prostate tumorigenesis [13]. BC200 RNA overexpression is correlated with the progression of breast cancer and has been proposed to be a new molecular marker for breast cancer [14]. Increased expression of MALAT-1 RNA indicates a worse clinical outcome in lung cancer patients [15]. Gupta et al. recently revealed an important role for HOTAIR in breast cancer metastasis. HOTAIR is highly induced (up to 2,000-fold) in breast cancer metastatic tissues [16]. In addition, overexpression of HOTAIR in primary breast tumors is a powerful predictor of eventual metastasis and death [16]. These studies provide evidence and support that lncRNAs may be involved in tumorigenesis and tumor progression.

However, research on the expression and function of lncRNAs in melanoma is still limited. In this study, we analyzed the expression profiles of 6 well documented metastasis-related lncRNAs in 3 pairs of primary melanoma and matched lymph node metastatic tissues using real-time quantitative RT-PCR. Further, we investigated the role of HOTAIR in melanoma cell motility and invasion. Our study provides novel insights into the role of lncRNAs in the metastatic progression of melanoma and identifies a potential new target for the treatment of metastatic melanoma. 

## 2. Materials and Methods

### 2.1. Tissue Samples

Three pairs of primary melanoma and matched lymph node metastatic tissues were obtained from the Department of Gastric Cancer and Soft Tissue Sarcoma Surgery at the Fudan University Shanghai Cancer Center. The protocol was approved by the Institutional Review Board of Fudan University. Patients enrolled in the study were provided with written informed consent. Fresh tissue samples were collected and cut into fragments <0.5 cm in any single dimension. The tissues were then immersed into 2 mL RNAlater (Ambion, Foster City, CA). All the tissue samples were frozen within 30 minutes after surgery and stored in liquid nitrogen until use. Tissue sections from each sample were reviewed and classified by a pathologist. 

### 2.2. Cell Culture

Human metastatic melanoma cell line A375 was obtained from the Typical Cell Culture Collection Committee of the Chinese Academy of Sciences. A375 cells were maintained in Dulbecco's modified Eagle's media (DMEM) supplemented with 10% fetal bovine serum (FBS).

### 2.3. Quantitative Reverse Transcriptase PCR (qRT-PCR) of Six lncRNAs

Total RNA was isolated from melanoma and lymph node metastatic tissues using the RNeasy kit (Qiagen, Grand Island, NY) according to the manufacturer's instructions. Reverse transcription (RT) reactions were performed with 1 *μ*g total RNA using a PrimeScript RT reagent kit (TaKaRa BIO, Shiga, Japan). Random hexamer primers were used in the RT reactions. Real-time qPCR was performed on a Bio-Rad CFX-96 real-time PCR system (Bio-Rad, Hercules, CA) using SYBR Premix DimerEraser kit (TaKaRa, Shiga, Japan). GAPDH was used as an endogenous control for the qRT-PCR reactions (TaKaRa, Shiga, Japan). All assays were performed in triplicates. The 2^−ΔΔCt^ method was used to calculate the expression of each lncRNA. All the reactions were carried out with strict compliance with the MIQE (Minimum Information about Quantitative Real-Time PCR Experiments) guidelines. Sequences of the real-time PCR primers are listed in Table 1.

### 2.4. siRNA Transfection

A375 cells (3 × 10^4^/well) were plated in 6-well plates overnight. Cells were then transfected with 50 nM nontargeting siRNA control (siControl), 50 nM siRNA against HOTAIR I (siHOTAIR I, SASI_Hs02_00380445), or siHOTAIR II (SASI_Hs02_00380446, Sigma Aldrich) for 24 h using Lipofectamine 2000 transfection reagent (Invitrogen, Grand Island, NY) according to the manufacturer's protocol. Following the transfections, A375 cells were then harvested for experiments as indicated.

### 2.5. Wound Healing Assay

A375 cells were transfected with siControl, siHOTAIR I, or siHOTAIR II and grew into confluent monolayers overnight. Scratches were generated with a sterile 200 *μ*L pipette tip. Cell migration towards the wounds was monitored at 0 hours and 24 hours using a light microscope.

### 2.6. Invasion Assay

Modified Boyden chamber assay was performed to assess cell invasion by using BioCoat Matrigel invasion chambers with 8 *μ*m pores (BD, Franklin Lakes, NJ). A375 cells (5 × 10^4^/chamber, in 100 *μ*L of serum-free DMEM) were added to the inserts of invasion chambers. FBS (10% in 600 *μ*L DMEM) was added as a chemoattractant in the lower chambers. After 24 hours of incubation, cells that had not penetrated the membranes were removed from the upper chambers with cotton swabs. Chamber membranes were fixed and stained using Diff-Quik Stain Set (Dade Behring Inc., Newark, DE) and examined under a bright-field microscope. Invasion was assessed by counting 6 fields per membrane (×20 objective).

### 2.7. In Situ Zymography

Glass coverslips were coated with 0.2 mg/mL Oregon green 488-conjugated gelatin (Invitrogen), cross-linked in 0.5% glutaraldehyde for 15 minutes at 4°C, and incubated with 5 mg/mL NaBH_4_ for 3 minutes. The coverslips were then sterilized with 70% ETOH for 15 minutes and incubated in serum-free media for 1 hour at 37°C. A375 cells transfected with siRNA-NS or siHOTAIR (I and II) were plated on gelatin-coated coverslips, incubated at 37°C for 24 hours, and processed by fluorescence microscopy procedures. Cell morphology was photographed under a light microscope.

### 2.8. Statistical Analysis

Statistical significances between groups were determined by two-tailed Student's *t*-test. For paired melanoma tissues, the difference of lncRNA expression was evaluated with Wilcoxon matched pairs signed ranks test. *P* < 0.05 was considered statistically significant.

## 3. Results

### 3.1. LncRNA Expression in Melanoma and Matched Lymph Node Metastatic Tissues

**Figure 1:**
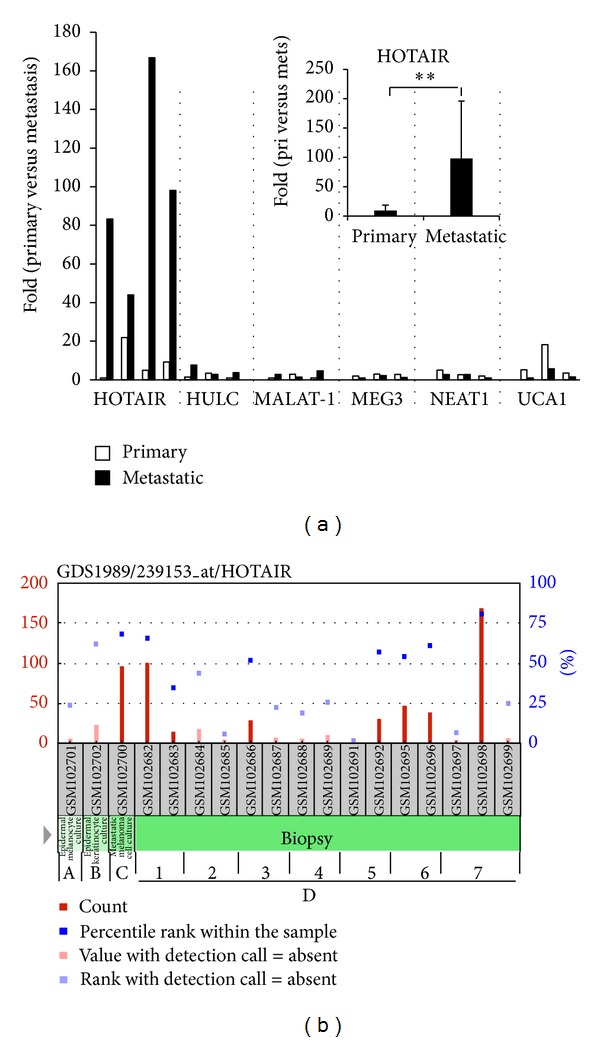
HOTAIR is upregulated in lymph node metastasis of melanoma. (a) Expression profiles of lncRNAs in melanoma and matched lymph node metastatic tissues. Quantitative RT-PCR was performed on 6 lncRNAs in melanoma and their matched metastatic tissue samples. HOTAIR expression was summarized in the insert. **P < 0.01. (b) GEO analysis of HOTAIR in 4 types of specimens and several disease states related to melanoma. The image was adapted from the GEO website. (A) Epidermal melanocyte culture; (B) epidermal keratinocyte culture; (C) metastatic melanoma cell culture; (D) biopsy. (1) Normal; (2) benign nevus; (3) atypical nevus; (4) melanoma in situ; (5) vertical growth phase melanoma; (6) metastatic growth phase melanoma; (7) lymph node metastasis.

Expression profiles of 6 lncRNAs that have been found to be associated with cancer or metastasis [17] were examined by qRT-PCR in 3 pairs of primary melanoma and matched lymph node metastatic tissues. We observed that most of the lncRNAs (5 out of 6) were differentially regulated in melanomas versus matched metastatic tissues (Figure 1(a)). Among them, HOTAIR was significantly overexpressed in metastatic lymph nodes compared to matched primary melanoma (*P* < 0.01) (Figure 1(a)). 

Further, data mining of publically available gene profiling database Gene Expression Omnibus (GEO) showed that HOTAIR expression is markedly higher in melanomas compared with nontumor tissues, and the highest expression was observed in tumors spread to regional lymph nodes (Figure 1(b)), suggesting that HOTAIR expression is linked to the progression and metastasis of melanoma.

### 3.2. Knockdown of HOTAIR Suppressed Metastatic Melanoma A375 Cell Motility

**Figure 2:**
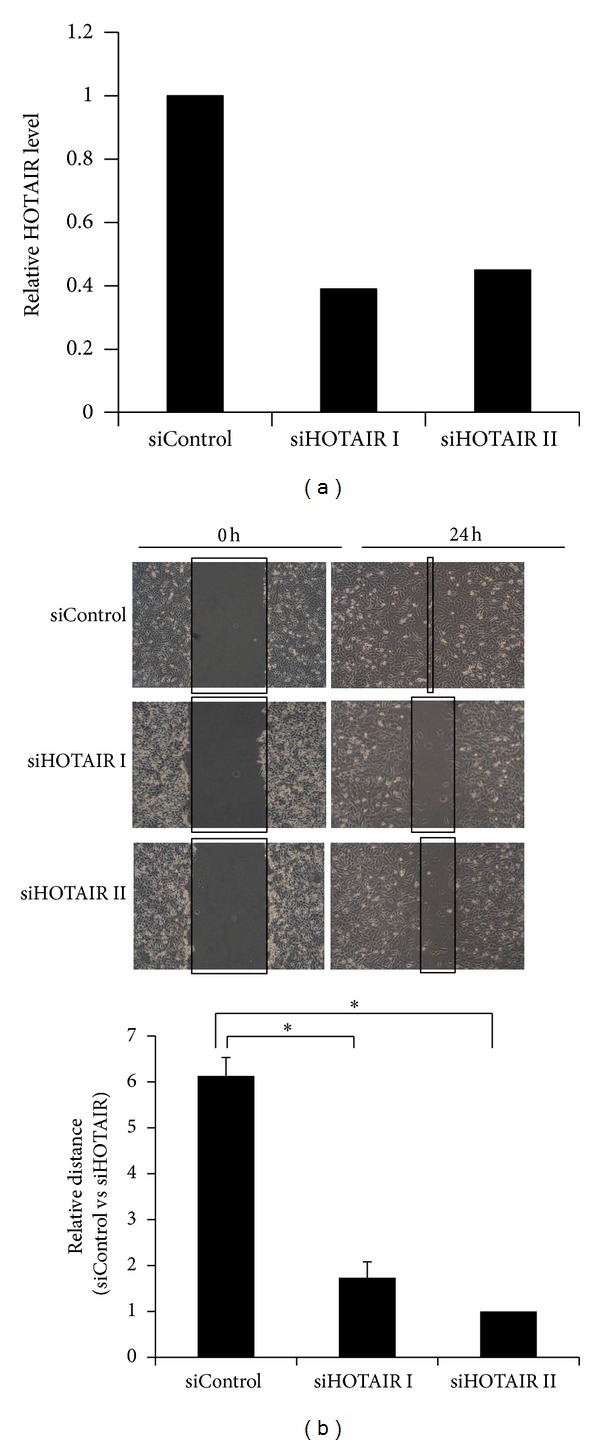
Knockdown of HOTAIR decreases melanoma A375 cell motility. (a) qRT-PCR analysis was performed to examine HOTAIR RNA levels in A375 cells transfected with siControl, siHOTAIR I, or siHOTAIR II. (b) Wounds were introduced by scratching confluent monolayers of A375 cells transfected with siControl, siHOTAIR I, or siHOTAIR II. Migration was monitored by light microscopy at 0 hours and 24 hours (upper panel). The widths of the gaps from 3 experiments were measured and the results are presented in a bar graph (lower panel). *P < 0.05.

Cell migration is an essential step in metastasis. Therefore, we next examined the ability of HOTAIR to affect cell motility using the scratch “wound” healing assays in human metastatic melanoma cell line A375. HOTAIR expression in A375 cells was knocked down by siRNAs and confirmed by real-time qPCR (Figure 2(a)). The wound healing assay showed that siControl-transfected A375 cells covered almost the entire damaged area by 24 hours (Figure 2(b)). In contrast, the wounded area was only partially covered by siHOTAIR-transfected cells after 24 hours of incubation (Figure 2(b)), suggesting that HOTAIR promotes melanoma cell motility.

### 3.3. Knockdown of HOTAIR Decreased A375 Cell Invasion

**Figure 3:**
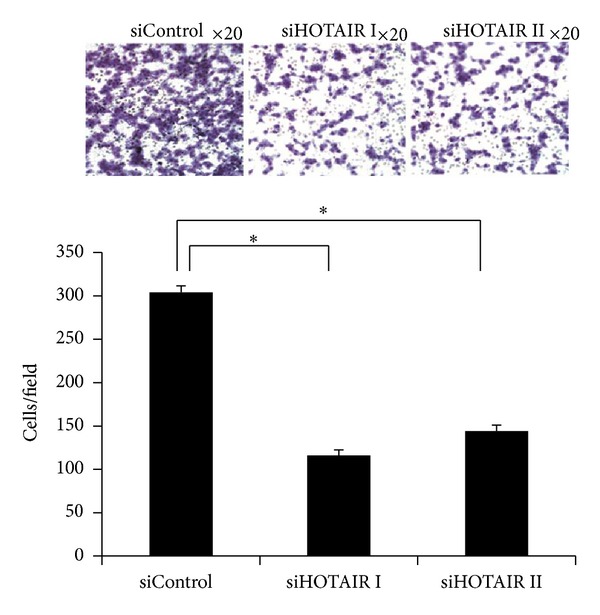
Knockdown of HOTAIR inhibits the invasion of melanoma A375 cells. Matrigel-based invasion assay was performed using modified Boyden chambers with 10% FBS as a chemoattractant. Representative images were presented. The cell numbers per field were counted and the results are summarized in a bar graph. *P < 0.05.

To further examine the involvement of HOTAIR in melanoma cell invasion, Matrigel-based Boyden chamber assay was performed. Knockdown of HOTAIR by siRNAs resulted in a ~3-fold reduction of the invasiveness of A375 cells (*P* < 0.01; Figure 3). Taken together, these data indicate that knockdown of HOTAIR inhibits in vitro parameters associated with metastasis including motility and invasion.

### 3.4. Degradation of Matrix In Situ Was Suppressed by Knockdown of HOTAIR

**Figure 4:**
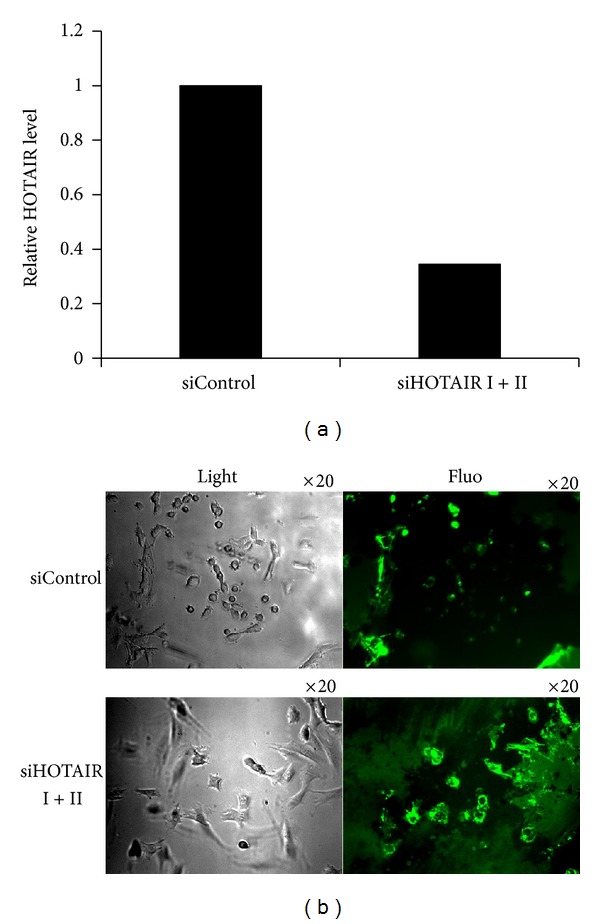
Degradation of matrix in situ is suppressed by knockdown of HOTAIR. (a) qRT-PCR analysis was performed to examine HOTAIR RNA levels in A375 cells transfected with siControl or siHOTAIR (I and II). (b) A375 cells were transfected with siControl or siHOTAIR (I and II) for 24 hours. In situ zymography was performed with Oregon green 488-conjugated gelatin. Cells were plated on gelatin matrix and incubated for 24 hours. The slides were observed by phase-contrast microscopy (left column), and gelatin degradation was visualized by fluorescence microscopy (right column).

Tumor cell invasion associated with metastasis requires both specialized cell migration and the ability to degrade basement membrane by secreted or membrane-bound proteases [18]. We sought to determine whether matrix metalloproteinase (MMPs), known to be upregulated in many metastatic tumors, might be responsible for HOTAIR-potentiated invasion. Gelatinase activity, indicating the activity of MMP-2 and MMP-9, was assessed by in situ zymography. The ability of A375 cells to degrade matrix in situ was markedly suppressed by siRNA-HOTAIR (I and II), as indicated by the reduced black holes representing matrix degradation (Figure 4(b)). This result suggests that HOTAIR promotes gelatinase activity in melanoma cells.

## 4. Discussion

Increasing studies provide evidence and support that ncRNAs are key factors in gene regulation and influence normal and cancer cell phenotypes [19–22].

LncRNAs belong to a novel class of ncRNA. They contain longer than 200 nucleotides and have no protein-coding capacity [23]. Several lncRNAs have been reported to control transcriptional alterations, implying that the difference in lncRNA profiling between normal and cancer cells is not just the secondary effect of cancer transformation [24]. On the contrary, lncRNAs are strongly associated with cancer progression [24]. However, little information regarding the expression profiles of lncRNAs in melanoma is available. Hence, in this study, we selected 6 well-documented lncRNAs associated with metastasis including MALAT1 [15, 16], HOTAIR [16], NEAT-1 [25], HULC [26], MEG-3 [27], and UCA1 [28] to evaluate their expression in primary melanoma and matched lymph node metastasis tissues. We observed that HOTAIR is the only consistently overexpressed lncRNA among the 6 lncRNAs in melanoma metastasis compared to matched primary tumors. Four other lncRNAs were differentially expressed in melanoma versus lymph node metastasis.

HOTAIR was initially identified as one of the 231 ncRNAs associated with human HOX loci [16]. HOTAIR promoted metastasis in breast cancer [16]. There is growing evidence that HOTAIR may have prometastasis activity in several cancer types, including breast [16], pancreatic [29], and hepatocellular carcinoma (HCC) [30]. Whether HOTAIR performs the same function in the progression of melanoma remains unknown. To understand the role of HOTAIR in melanoma progression, a series of in vitro assays were performed. Studies using wound healing assay demonstrate that knockdown of HOTAIR inhibits the migration of melanoma cells. The invasiveness of melanoma cells is also markedly suppressed by knocking down HOTAIR, as demonstrated by the Matrigel-based Boyden chamber assay. Recently, Geng et al. found that HOTAIR expression is elevated in HCC tumors compared with adjacent nontumor tissues [31]. HOTAIR expression is correlated with lymph node metastasis in HCC [31]. siRNA-mediated knockdown of HOTAIR in HCC cells was accompanied by a deduction in MMP-9, suggesting that MMP-9 may be involved in HOTAIR-mediated regulation of HCC progression [31]. As MMP-9 plays an important role in tumor metastasis, gelatinase activity (indicating the activity of MMP-2 and MMP-9) was assessed by in situ zymography, which is a unique technique for revealing proteolytic activity at specific sites in tissues or cell cultures. Results indicate that the degradation of matrix was suppressed by knockdown of HOTAIR, supporting the hypothesis that HOTAIR promotes the activity of MMP-9 and/or MMP-2. BRAF-activated noncoding RNA (BANCR) shows increased expression in melanoma [32]. Knockdown of BANCR reduced melanoma cell migration [32]. These data suggest that lncRNAs may play an important role in the progression of melanoma. Further research on lncRNA expression profiles is needed to define the impact of lncRNAs on the progress of melanoma.

Interestingly, in our study, the expression levels of lncRNA MALAT1 show no significant difference between primary melanoma and matched metastatic tissues. On the contrary, MALAT1 was documented by several studies to associate with metastasis in other cancer types [15]. Increased expression of MALAT1 was first observed in metastatic non-small cell lung cancer [15], followed by endometrial stromal sarcoma of the uterus [33], and more recently in six other types of cancer, including HCC, breast, pancreas, lung, colon, and prostate cancers [34]. Overall, these results suggest that the effect of lncRNAs on cancer progression may be cancer-type specific. 

In conclusion, we demonstrate that HOTAIR lncRNA is predominantly upregulated in lymph node metastasis tissues compared with primary melanoma. Knockdown of HOTAIR inhibits the motility and invasiveness of melanoma cells, and the latter is associated with decreased degradation of extracellular matrix. Although further mechanistic investigation into the regulation of metastasis by HOTAIR is necessary, the observed prometastatic activity of HOTAIR in multiple preclinical model systems supports HOTAIR to be a potential target for melanoma metastasis therapy.

## Figures and Tables

**Table 1:** Nucleotide sequence of primers for lncRNA qPCR.

IncRNA	Sequence
HOTAIR-F	5′-CAGTGGGGAACTCTGACTCG-3′
HOTAIR-R	5′-GTGCCTGGTGCTCTCTTACC-3′
HULC-F	5′-TCATGATGGAATTGGAGCCTT-3′
HULC-R	5′-CTCTTCCTGGCTTGCAGATTG-3′
MALAT1-F	5′-TAGGAAGACAGCAGCAGACAGG-3′
MALAT1-R	5′-TTGCTCGCTTGCTCCTCAGT-3′
MEG3-F	5′-GCCAAGCTTCTTGAAAGGCC-3′
MEG3-R	5′-TTCCACGGAGTAGAGCGAGTC-3′
NEAT1-F	5′-TGGCTAGCTCAGGGCTTCAG-3′
NEAT1-R	5′-TCTCCTTGCCAAGCTTCCTTC-3′
UCA1/CDUR-F	5′-CATGCTTGACACTTGGTGCC-3′
UCA1/CDUR-R	5′-GGTCGCAGGTGGATCTCTTC-3′
